# Books of Interest to Nurses

**Published:** 1920-09-25

**Authors:** 


### BOOKS OF INTEREST TO NURSES.

**The Nursing of Chronic Patients.** By Eleanor C. Barton, R.R.C., Matron, Chelsea Infirmary. (London: The Scientific Press, Ltd, 28/29 Southampton Street, Strand, W.C. 2. Price 1s. 3d. net post free.)

If it were possible to ascertain from the nursing profession what branch of nursing is the most popular, it would be found probably that surgical nursing attracts the interest of far the larger number. Yet most experienced nurses would agree that the successful medical nurse has achieved an ideal which commands the admiration of all. The reason for the preference is perhaps because the acquisition of the technical skill demanded by surgery offers an obtrusive interest, whereas in the nursing of medical cases, more especially chronic cases, the interest is apparent only to the few who seek it. Intuition, sympathy, and love of human nature and patience reveal unlimited interest in cases which the superficial might regard as tiresome and unattractive.

Miss Barton deals with the subject in an able and appealing manner; if she succeeds only in arousing a more sympathetic regard for this branch of nursing, Miss Barton will have done a valuable service for the nursing profession. Apart from this aspect of the matter, however, the chapters on the various types of cases so frequently met with in the poor-law infirmaries are highly instructive and full of that wisdom and good sense which are the outcome of mature experience.

**Simple Beginnings in the Training of Mentally Defective Children.** By Margaret Macdowall. (London: Local Government Press, 27 Furnival Street, E.C. 3s. 6d.)

An unfortunate result of our habit of putting people in categories is that we are apt to leave them there. Of late years the feeble-minded children have been sorted out from amongst others with great benefit to both kinds. But within the category are many degrees and varied kinds of affliction. The microcephalic child, the "mongol" and the merely nervous and undeveloped are not on the same level, all may be improved, some to the point of becoming useful and self-respecting citizens. In a touching and most hope-inspiring little book Miss Margaret Macdowall has now given for the encouragement of others some results of her own experience and practical suggestions for treatment and for teaching, even to simple lessons in the rudiments of cookery and laundry work. In her little school in Sussex, Miss Macdowall and her devoted helpers have recognised no impossibilities, though love and wisdom, born of daily watchfulness and effort, must add infinite patience for the perfecting of the teacher. Years of effort may be needed to turn the dribbling imbecile into an almost normal human being, yet the victory is worth while. First the mere power to use the lip muscles and close the flaccid mouth may have to be gained by such means as blowing little tin trumpets, and the ability to bite solid food attained by learning to gnaw a rubber ring. One child of seven with useless legs was first placed in a low chair to learn to press the foot-soles on a floor, then hung in a higher chair from which the seat was removed, with toys hung round within reach of its hands. Then followed a walking-machine and the pushing of a toy cart. Miss Macdowall finds the Montessori appliances useful, but has evolved others of her own, wooden discs for threading as an introduction to beads and bamboos, receivers for balls which teach form, number, colour, and develop the finger muscles. Every case will differ in the mental as well as the physical needs. "*He* may copy easily, while years of patient teaching cannot get anything done by him from dictation, whereas *she* will remember directions, correct them, and produce a decent 'r' when told, but the same years of patience will not get her to see clearly enough to copy unaided." An interesting point is that inability to articulate may have its compensations in better power of listening and observing. Miss Macdowall's view that religious ideas, if rightly presented, are fit subjects for teaching is very welcome. It may be hoped that her little book will awaken a sense of vocation in some women eager to help in national reconstruction.

**A Handbook of Midwifery.** By Comyns Berkeley, M.D., F.R.C.P. Fifth edition. (Cassell and Co. Pp. 550. Price 7s. 6d. net.)

This excellent handbook for midwives, maternity nurses, and obstetric dressers has been reviewed before in this journal, and has evidently obtained the wide popularity which its merits deserve. In the present edition, which has been thoroughly revised, the sections on breast-feeding and artificial feeding have been re-written. Unfortunately, there has crept in a curious statement, doubtless a misprint, which may mislead those for whom the book is designed. On p. 404 (dealing with breast-feeding) it is stated that the newborn child is to be fed four times daily for the first ten days of its life: for *ten* days, it may be supposed, *two* days should be read. In view of the widespread popularity and authoritative reputation which the book enjoys, it is important that the correction should be made as soon as possible. In all other respects the handbook fully maintains the high standard of the past.

